# Neuroimaging Guided Transcranial Electrical Stimulation in Enhancing Surgical Skill Acquisition. Comment on Hung et al. The Efficacy of Transcranial Direct Current Stimulation in Enhancing Surgical Skill Acquisition: A Preliminary Meta-Analysis of Randomized Controlled Trials. *Brain Sci.* 2021, *11*, 707

**DOI:** 10.3390/brainsci11081078

**Published:** 2021-08-18

**Authors:** Pushpinder Walia, Kavya Narendra Kumar, Anirban Dutta

**Affiliations:** 1 Department of Mechanical and Aerospace Engineering, University at Buffalo, Buffalo, NY 14260, USA; pwalia@buffalo.edu; 2 Department of Biomedical Engineering, University at Buffalo, Buffalo, NY 14260, USA; kavyanar@buffalo.edu

**Keywords:** surgical skill acquisition, portable neuroimaging, functional near-infrared spectroscopy, transcranial electrical stimulation

## Abstract

Surgical skill acquisition may be facilitated with a safe application of transcranial direct current stimulation (tDCS). A preliminary meta-analysis of randomized control trials showed that tDCS was associated with significantly better improvement in surgical performance than the sham control; however, meta-analysis does not address the mechanistic understanding. It is known from skill learning studies that the hierarchy of cognitive control shows a rostrocaudal axis in the frontal lobe where a shift from posterior to anterior is postulated to mediate progressively abstract, higher-order control. Therefore, optimizing the transcranial electrical stimulation to target surgical task-related brain activation at different stages of motor learning may provide the causal link to the learning behavior. This comment paper presents the computational approach for neuroimaging guided tDCS based on open-source software pipelines and an open-data of functional near-infrared spectroscopy (fNIRS) for complex motor tasks. We performed an fNIRS-based cortical activation analysis using AtlasViewer software that was used as the target for tDCS of the motor complexity-related brain regions using ROAST software. For future studies on surgical skill training, it is postulated that the higher complexity laparoscopic suturing with intracorporeal knot tying task may result in more robust activation of the motor complexity-related brain areas when compared to the lower complexity laparoscopic tasks.

Surgical skill acquisition may be facilitated with a safe application of transcranial electrical stimulation (tES) [1]. Transcranial direct current stimulation (tDCS), a tES modality, has been shown to facilitate surgical skill learning when applied to cortical targets, including the primary motor cortex [2,3], the supplementary motor area [2], and the prefrontal cortex [4]. Prior work has shown that tDCS facilitated complex motor tasks performed during surgical skill training, including laparoscopic technical skills training [5] and tumor resection in neurosurgery [6]. These results are in accordance with several human studies following initial studies by Nitsche and Paulus [7] that have shown the beneficial effect of tDCS on motor learning and suggested that tDCS may play an adjuvant role in combination with motor training in health and disease [8,9]. Its mechanism of action is by enhancing cortical excitability, which enhances the probability of learning-related processes [10]. Here, neuroplasticity is the ability of the central nervous system to respond to intrinsic or extrinsic stimuli by reorganizing its structure, function, and connections. Recently, Hung et al. [11] presented the first preliminary meta-analysis of randomized control trials that showed that tDCS was associated with significantly better improvement in surgical performance than the sham control. Hung et al. [11] found that tDCS over the bilateral prefrontal cortex (PFC) and the primary motor cortex (M1) were both associated with significantly better improvements in surgical performance. Since complex motor tasks [12] involve motor control and attention-related brain areas, it is expected that both the PFC and M1 stimulation may facilitate task performance. However, meta-analysis does not address the mechanistic understanding, and Hung et al. [11] did not provide further evidence on the mechanism of tDCS action on the learning behavior. Since the tDCS effects on the learning behavior were not analyzed separately at the three stages of learning motor skills: a cognitive phase, an associative phase, and an autonomous phase [13], so all the investigated tDCS montages were found to be facilitatory when lumped together in this preliminary meta-analysis. Especially, the slowest learning stage is associated with activity in the cerebellum [14] that can be facilitated with cerebellar tDCS [15,16]. Then, in surgical skill acquisition, the investigation of the brain-behavior relationship in terms of perception-action coupling [17] can provide insights in to the learning process.

Fundamentals of Laparoscopic Surgery (FLS) is a pre-requisite for board certification in general surgery in the USA, where five psychomotor tasks with increasing task complexity are used: (i) pegboard transfers, (ii) pattern cutting, (iii) placement of a ligating loop, (iv) suturing with extracorporeal knot tying and (v) suturing with intracorporeal knot tying. During skill learning, the hierarchy of cognitive control shows a rostrocaudal axis in the frontal lobe [18], where a shift from posterior to anterior is postulated to mediate progressively abstract, higher-order control. Here, the PFC can be divided into functional subregions where medial PFC is related to abstract second-order relationships, including autobiographical memory recall and decision making, while the dorsolateral and ventrolateral PFC can be related to feature extraction and formation of first-order relationships [19,20,21]. Therefore, it can be postulated that the novices will have primarily lateral PFC activation at the initial skill learning stage when the externally generated information has to be evaluated, and then, a shift from posterior-to-anterior PFC will underpin “automaticity” in cognitive control. This evolution of brain activation during visuomotor learning [22] is related to the changes in the brain network where gain in early performance has been shown to rely strongly on the prefrontal-caudate interactions. Therefore, fNIRS-guided tDCS is proposed to target subject-specific endogenous PFC activation related to the prefrontal-caudate network-level mechanisms to be effective [23]. Here, the initial skill learning stage during FLS pattern cutting task, with primarily PFC activation [24], is postulated to require tDCS of the subject-specific activity in the functional PFC subdomains. Moreover, an intact action-perception coupling, that is relevant for surgical skill acquisition, has been shown to depend on the integrity of the cerebellum [25]. Christensen et al. [25] investigated the action-perception coupling based on the effect of action execution on action-perception that is postulated to be crucial during surgical skill acquisition in physical as well as virtual simulators [26]. Human functional neuroimaging has shown segregated fronto-cerebellar circuits [27], e.g., dorsolateral PFC (DLPFC)-correlated activity was shown to span cerebellar Crus I/II lobules in its lateral and ventral extent while medial PFC (MPFC)-correlated activity spanned cerebellar Crus I lobule. Crus I preferentially correlated with MPFC, while Crus II preferentially correlated with DLPFC that can be targeted with tES in an age-specific manner [28] where posterolateral cerebellum and cerebrum is feasible for fNIRS monitoring [29]. Then, fNIRS-based monitoring of the functional brain connectivity may be crucial to capture neural correlates of learning during surgical training where wavelet coherence between the medial PFC and the supplementary motor area was found lower in experts than untrained subjects in FLS physical simulators [30]—a postulated marker of skill dexterity.

Nemani et al. [24] demonstrated that the FLS pattern cutting task-related activation of the PFC and M1 change at different stages of motor learning. During FLS bimanual pattern cutting task [24], the PFC activation decreased with increased motor skill proficiency while fine motor control-related brain regions showed increased activation in the experts. Here, the skilled trainee population demonstrated a significantly different brain response after day 7 when compared to the first day of training. Then, in a bimanual pattern cutting study with tDCS of the M1 along with portable neuroimaging [3], Gao et al. observed that the M1 tDCS effect on the performance error was significant (*p* < 0.001; t-test when normally distributed or Mann–Whitney U test when not) after day 7 when compared to the sham group. Here, a delayed effect of M1 tDCS after day 7 was found that is postulated to be related to the emergence of M1 activation that was significant (*p* < 0.001) only during the latter learning stage (day 7–12) when compared to the initial learning stage (day 2–6) [3]. This is expected from known in vivo effects of tDCS that do not change the firing rates of the cortical neurons [31] but modulate endogenous task-specific brain activity [32], so neuroimaging can provide the “target” cortical activation related to endogenous task-specific brain activity. This “target” cortical activation for tDCS can be a part of the central-executive [33] and the motor [34] network that are relevant in motor skill learning; however, they have different roles (e.g., cognitive control [35], motor control [36]), relevant at different stages of skill learning [21]. Therefore, we postulate the importance of the individualized tDCS electrode montage to target surgical task-related brain activation at various stages of motor learning that can be measured with portable neuroimaging, including fNIRS [24] and electroencephalography (EEG) [37]. Then, combined fNIRS-EEG can also be used to monitor individual brain responses to tDCS [38].

A recent study demonstrated the feasibility of prefrontal tDCS to facilitate early-phase surgical-skill acquisition [4]. This is expected since activation in the PFC is expected in the early phase of skill acquisition when attention and working memory are required to actively monitor targets in the environment until ‘automaticity’ is achieved. Ashcroft et al. [4] used a one-size-fits-all approach with an anode over F3 (10/10 EEG montage) and cathode over F4 delivering 2mA tDCS for 15 min and found an improved performance score in an open knot tying task (three repeated blocks) when compared to sham tDCS (*p* = 0.002). Here, F3–F4 tDCS was postulated to target the associative network, including the dorsolateral PFC primarily. However, more complex FLS tasks, e.g., suturing with intracorporeal knot tying, will require attentional control (for feature extraction from surgical field) in the inferior frontal gyrus (IFG) [39] and polymodal processing in the ventral premotor cortex (PMv) [40,41] that was shown by Walia et al. [42]—including inter-individual differences in novices that necessitates individual fNIRS monitoring. Since fNIRS has been shown to be feasible during surgical task performance [26], AtlasViewer [43,44] in Matlab (Mathworks Inc., Natick, MA, USA) can be used to determine the task-related cortical activation based on the hemodynamic response function. Here, Walia et al. [42] results partially aligned with Li et al. [43], who also found pars opercularis IFG/PMv to be one of the motor complexity sensitive brain regions. However, Walia et al. [42] found left lateralized PFC activation in a group right-handed subjects that may be related to the short frontal lobe connections of the human brain [45]—needs further investigation based on functional connectivity analysis. Then, the centroid of the cortical activation was found by calculating the average position in the activation “mass” weighted by the image intensity [44]. This centroid was used after mapping to the MNI-152 standard head (individualized head model was created from structural MRI in SPM software—https://www.fil.ion.ucl.ac.uk/spm/: accessed on 1 June 2021) for optimizing tDCS using the open-source ROAST pipeline [46]. Figure 1A shows the block diagram for subject-specific neuroimaging-guided tES. To test the feasibility of this approach, we used the open-access fNIRS dataset from Li et al. [43]. Figure 1B shows the right hemisphere brain activation during the execution of the motor complexity task that resulted in more robust activation of the pars opercularis IFG, PMv, and inferior parietal lobule due to postulated increased motor preparation and planning [43]. Then, Figure 1C shows the neuroimaging-guided tES where a single function, “roast_target,” was used for the optimization under criteria, maximal-focality using the “MNI152”-based lead-field matrix and default parameters. For inter-hemispheric comparison, Figure 1D shows the left hemisphere brain activation during the execution of the motor complexity task. For future studies on neuroimaging-guided tES facilitated FLS skill training, it is postulated that the higher-complexity FLS suturing with intracorporeal knot tying task may result in stronger brain activation of the motor complexity related areas, possibly underpinned by the frontal ‘aslant’ tract [45], than the lower-complexity FLS tasks in our prior works [24]. Here, portable neuroimaging-based cortical activation and functional connectivity [26] estimates can guide tES application, i.e., portable neuroimaging-guided tES, to facilitate surgical skill acquisition and monitoring of the brain response to tES [47,48] for adequate dosing of the task-related brain areas.

## Figures and Tables

**Figure 1:**
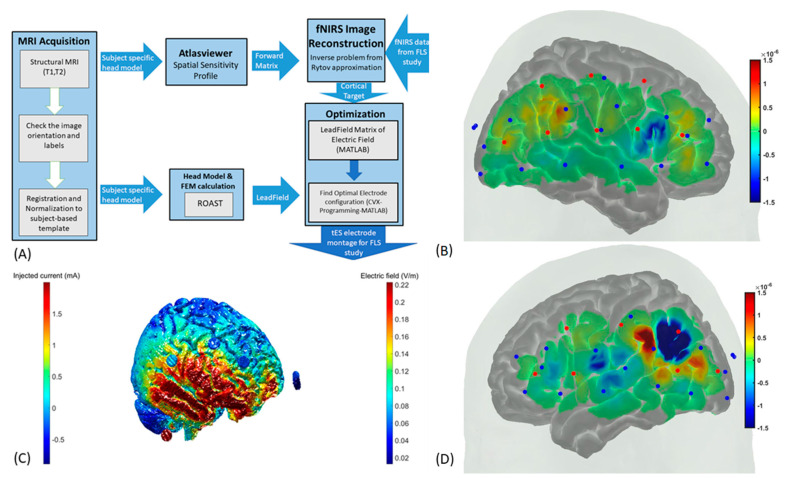
Neuroimaging-guided transcranial electrical stimulation. (**A**) An individualized head model can be created from structural MRI in SPM software (https://www.fil.ion.ucl.ac.uk/spm/: accessed on 1 June 2021) that can be used in AtlasViewer and ROAST to create a subject-specific forward matrix and lead field, respectively. AtlasViewer uses the forward matrix to estimate the cortical activation from task-related fNIRS data, which can be used as the cortical target for the electrode optimization in ROAST based on the lead field. (**B**) Right hemisphere cortical activation from AtlasViewer during the execution of a complex motor task (red dots show the sources and blue dots show the detectors) from Li et al. [43]. (**C**) Right hemisphere cortical activation during the execution of a complex motor task (from Li et al. [43]) was targeted with the ROAST-optimized electrode montage as shown by the electric field distribution (V/m). (**D**) Left hemisphere cortical activation from AtlasViewer during the execution of a complex motor task from Li et al. [43].

## Data Availability

Data is available at https://openfnirs.org/data/ (accessed on 1 June 2021).
